# High-Efficiency Microsatellite-Using Super-Resolution Algorithm Based on the Multi-Modality Super-CMOS Sensor

**DOI:** 10.3390/s20144019

**Published:** 2020-07-20

**Authors:** Ke Zhang, Cankun Yang, Xiaojuan Li, Chunping Zhou, Ruofei Zhong

**Affiliations:** 1 Key Laboratory of 3D Information Acquisition and Application, MOE, Capital Normal University, Beijing 100048, China; zhangke017@cnu.edu.cn (K.Z.); lixiaojuan@cnu.edu.cn (X.L.); zrf@cnu.edu.cn (R.Z.); 2 Engineering Research Centre of Spatial Information Technology, MOE, Capital Normal University, Beijing 100048, China; 3 The Institute of Remote Sensing, Beijing 100011, China; zcp1688@sina.com

**Keywords:** multi-modality, rotatable sensor, microsatellite spatial resolution, oblique sampling mode, super-resolution, algorithm engineering

## Abstract

To realize the application of super-resolution technology from theory to practice, and to improve microsatellite spatial resolution, we propose a special super-resolution algorithm based on the multi-modality super-CMOS sensor which can adapt to the limited operation capacity of microsatellite computers. First, we designed an oblique sampling mode with the sensor rotated at an angle of $26.56^{\circ }\left(\arctan\frac{1}{2}\right)$ to obtain high overlap ratio images with sub-pixel displacement. Secondly, the proposed super-resolution algorithm was applied to reconstruct the final high-resolution image. Because the satellite equipped with this sensor is scheduled to be launched this year, we also designed the simulation mode of conventional sampling and the oblique sampling of the sensor to obtain the comparison and experimental data. Lastly, we evaluated the super-resolution quality of images, the effectiveness, the practicality, and the efficiency of the algorithm. The results of the experiments showed that the satellite-using super-resolution algorithm combined with multi-modality super-CMOS sensor oblique-mode sampling can increase the spatial resolution of an image by about 2 times. The algorithm is simple and highly efficient, and can realize the super-resolution reconstruction of two remote-sensing images within 0.713 s, which has good performance on the microsatellite.

## 1. Introduction

Since the 20th century, issues such as resources, environment, disasters, and national security have become the main problems in the development of human society. As one of the important means to solve these problems, space-to-earth observation technology has received much more attention from all countries, which has promoted the rapid development of aerospace and remote-sensing technology. At present, refined remote sensing has become the main direction of the development of the current remote-sensing field.

Generally speaking, there are mainly two ways to improve satellite image resolution. One is known as the “hardware way”. Since 1970, CCD (Charge-coupled Device) and CMOS (Complementary Metal Oxide Semiconductor) image sensors have been widely used in satellite remote sensing. The most direct method is to reduce the size of the photosensitive unit by improving the sensor fabrication process to increase the number of pixels in the image. Increasing camera focal length is another “hardware way”. It can reduce the instantaneous field of view while ground sampling, but it results in more processing difficulty and higher manufacturing cost of optical parts. Due to the limitation of technology level and image data transmission rate, the resolution of images obtained by high-resolution CCD camera is very limited [1]. Considering these factors, the technology of image super-resolution has been proposed, which is called the “software way”. To achieve the effects of improving image resolution, highlight feature details, and improve image quality, the super-resolution technology increases the number of image pixels by a variety of ways such as information fusion, prior constraint, and so on [2]. By dealing with images, it can break the limitations of current imaging devices. However, this technology has limited by application scenarios, and the improvement effect is susceptible to the redundancy of complementary information, which restricts the wide application of super-resolution technology in the field of remote sensing.

Therefore, we present a method of combining hardware equipment with software algorithm to improve the resolution of microsatellite imaging. The authors designed a hardware equipment which is known as multi-modality super-CMOS sensor, or MS-CMOS for short. Its sampling mode can effectively increase the sampling density in both the satellite flight direction and the perpendicular direction and provide the best data for the satellite-using super-resolution algorithm. For the software algorithm, which is based on the sub-pixel super-resolution theories, we proposed a special super-resolution algorithm using MS-CMOS data which can adapt to the limited operation capacity of microsatellite computers.

This paper is organized as follows: some related works are mentioned in Section 2. The multi-modality super-CMOS sensor is introduced in Section 3. The data-simulation method is proposed in Section 4. The super-resolution algorithm based on the oblique sampling model is proposed in Section 5. The experiments and discussion are shown in Section 6. Section 7 shows the conclusion.

## 2. Techniques for Improving the Resolution of Remote-Sensing Images

### 2.1. Satellite–Ground Combined Super-Resolution Technology

Super-resolution (SR) is the process of obtaining high-resolution (HR) images from one or more low-resolution (LR) images. However, there is not a general SR method or technology that can solve all the SR problems. The SR algorithm and parameters should be selected according to different application scenarios and conditions. Because of the limitation of system design and the complexity of the imaging environment, the spatial resolution of the image is restricted. Therefore, scholars have proposed a theory which is to combine the hardware and software, satellite, and ground to reduce the cost of human resources and material resources, and effectively improve image resolution. This method can realize multi-image fusion and improve the resolution by designing the special arrangement of multi-sensor and bypassing the image registration and correction.

French Centre National d’Etudes Spatiales (CNES) proposed a CCD arrangement method to increase the spatial resolution of SPOT5 satellite sensor. The SPOT5 satellite SR mode, SUPERMODE sampling and HIPERMODE sampling were proposed. The two sampling methods used the customized two rows CCD device with 0.5 pixel staggered in the linear array direction and 3.5 pixel staggered in the satellite flight direction [3] (pp. 318–320). HIPERMODE sampling method realized the fusion of two 5 m resolution images to obtain one 2.5~3 m HR image. The concept of STAGGERED ARRAY was proposed by the Hot Spot Recognition Sensors (HSRS) which was developed by Deutsches Zentrum für Luft und Raumfahrt (DLR) [4]. The core of the HSRS sensor was a staggered 512 × 2 double infrared ray array. The dislocation arrangement was specific: stagger 0.5 pixels in the array direction and stagger 2 whole pixels in the vertical array direction to obtain multiple images with pixel offset. After processing, the spatial resolution of the camera can be improved. The Leica ADS40 line scanner system consisted of two staggered CCD linear arrays. The ADS40 sampled at twice the frequency in the flight direction to obtain multiple images with half-pixel offset [5] (p. 565). In addition, a method of recovering HR images from interlaced lines was proposed, which can improve the spatial resolution of the camera [6]. Israel had developed a series of EROS (Earth Remote Observation System) satellites. The outstanding feature of this satellite was that it had the ability of single orbit imaging. This function allowed repeated sampling of the same area by tilting the camera. The resolution of EROS-B1 satellite can reach 0.85 m, and the over-sampling mode can reach a resolution of 0.5 m [7]. Nigerian satellite 2 was a small satellite with a spatial resolution of 2.5 m. The satellite design allowed intentional yaw, which reduced the width of the pixel’s ground track to achieve image SR [8]. SkySat series satellites used the method of surface array push-frame combined with ground processing, by the sequence image registration and SR algorithm processing on the ground, the image resolution was improved from 1.3 m to 0.9 m, and sub-meter imaging was realized [9,10]. Moreover, a satellite platform was designed to capture images with overlapping parts continuously to solve the problem of poor imaging quality caused by the limited payload of Nanosatellite [11]. They used the normalized cross-correlation method to determine the sub-pixel transformation between images, laying a foundation for CubeSats to achieve SR.

In China, Zhou, C. P. [12] (pp. 1–27), [13] proposed the satellite oblique-mode sampling method, which combined satellite hardware with ground-based image processing software to improve the spatial resolution. By tilting the linear array detector 45° and adjusting the integration time, Zhou, F. [14] improved the resolution of the sampled image by about 1.64 times compared with that before rotation.

### 2.2. Software-Hardware Combined Sub-Pixel Super-Resolution Algorithm

The sub-pixel imaging is a method to obtain multiple LR images with mutual dislocation information of the same target first, and then extract the redundant information in the shifted image to improve the resolution, which is consistent with the core of two technologies mentioned above. The proposed SR algorithm is based on the similar idea, it is necessary to discuss the sub-pixel SR algorithm in this section.

Since the last century, there had existed resolution improvement methods which used displacement images. Aizawa [15] (pp. 289–292) proposed a method to improve the resolution of a single CCD sensor by software synthesis after displacement imaging using CCD. Hardie [16] used micro-scanning to reduce the interference noise generated by image sub-sampling, to realize the synthesis of multiple digital images and improve the resolution. 

In the remote-sensing imaging system, An [17] used adjacent layers of optical fibers arranged into hexagonal patterns to obtain multiple images of sub-pixel dislocation, designed the interpolation function of image fusion, and improved the spatial resolution. Wu [18] designed a rotary micro-scanner, which can effectively improve the spatial resolution of infrared images and improve the function value of the system. Sun and Yu [19] believed that sub-pixel scanning via mobile sensors would be the key to SR reconstruction. They proposed a sur-pixel scanning method with low processing difficulty, which verified its advantages in engineering. Li [20] designed a registration method based on the sub-pixel displacement estimation to artificially improve the computational efficiency of image SR reconstruction, and verified the effectiveness of the method through experiments. Zhao [21] used a dynamic single-pixel camera to obtain sequential images with sub-pixel displacement and reconstructed the final HR image using wiener filter. Compared with the traditional single-pixel imaging system, this method had better spatial resolution and SNR (Signal Noise Ratio). Zhang [22] had designed a controllable sub-pixel micro-scanning infrared optical system which can achieve any step size of sampling, with the purpose of obtaining sub-pixel LR images, realize the de-noising and SR reconstruction of infrared images at the same time. Wang [23] used Risley prisms to design a low cost and high-efficiency method that can improve the image resolution of the imaging system when the resolution is limited by the pixel size. They established the mathematical model of SR reconstruction for single-camera sub-pixel imaging.

### 2.3. Remote-Sensing Image Post-Processing Technology

Remote-sensing image post-processing technology refers to the use of multiple LR images of the same scene in multiple time phases, combined with image registration and image SR algorithms, to achieve the SR reconstruction of remote-sensing image. Because the remote-sensing data has multi-phase, multi-angle, and multi-band complementary information, it can greatly meet the needs of SR reconstruction for complementary information. The traditional SR reconstruction of remote-sensing image mainly uses multi-temporal image data, in fact, the idea of SR reconstruction originated from processing multi-temporal Landsat remote-sensing image. By applying the theory of using single or multiple satellites to post-process and synthesize multiple images of the same target to improve image resolution, China’s Qian Xuesen Laboratory of Space Technology had broken through the key techniques of image registration and reconstruction of remote-sensing images SR processing, and carried out an experiment to improve the resolution of GaoFen-4 images [24,25].

For remote-sensing satellites, the theoretical research and academic discussion of image SR algorithm do not have the basis of engineering temporarily, which is not enough to achieve the purpose of improving the spatial resolution. There are many problems in the hardware design of satellite imaging sensor to improve the spatial resolution, such as the difficulty of realization, the long period of research and development, and the high cost of manufacture. Many image SR technologies, including deep learning, have a short application time in the field of remote sensing. The common idea is to conduct data fusion for LR images, which is prone to generate false information and requires the support of high-performance computer equipment, the processing capacity of satellite computer cannot meet the requirements. The combination of hardware design and software algorithm has become a new idea to improve the resolution of remote-sensing image for its low cost and convenience.

## 3. Multi-Modality Super-CMOS Sensor

### 3.1. Structure and Imaging Model

In 1969, a CCD Image Sensor was developed at the Alcatel-Lucent Bell Labs. The CCD Image Sensor has many advantages such as small volume, high resolution, high sensitivity, and so on. The research of the sensor in this paper choses CMOS device which has the same origin with CCD electric coupling device, but the price is lower.

**Figure 1:**
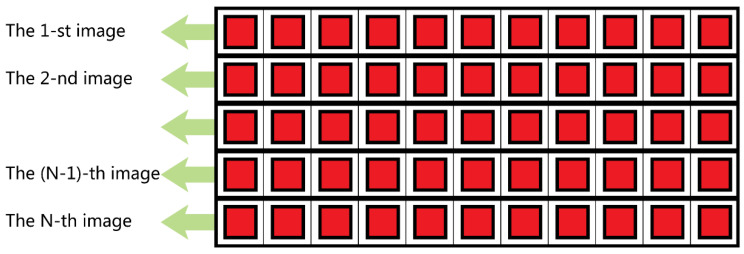
The diagram of MS-CMOS structure.

We name this special sensor as multi-modality super-CMOS sensor, MS-CMOS for short. Its hardware structure is similar to a planar array CCD sensor, as shown in Figure 1. The imaging and output of MS-CMOS are similar to a linear array CCD sensor. Each row that forms the MS-CMOS is separately controlled, images simultaneously, and outputs independently.

### 3.2. Imaging Model and Sampling Model

Unlike the traditional push-broom or push-frame imaging along the satellite flight direction, MS-CMOS can achieve different types of imaging by controlling parameters such as CMOS row number, rotation angle, and so on. The MS-CMOS imaging model can be represented by the function (1):

(1) $$I_{m}=F\left(N,M,\alpha ,T_{N},t_{i}\right)$$

In this function:

$I_{m}$: MS-CMOS imaging model,

*N*: number of line CMOS sensors number(vertical),

*M*: pixel number of line CMOS sensor(lateral),

α: CMOS sensor rotation angle,

$T_{N}$: sampling time of each line CMOS sensor,

$t_{i}$: integration time.

When *N* = 1, MS-CMOS can realize linear array CMOS sensor function.

**Figure 2:**
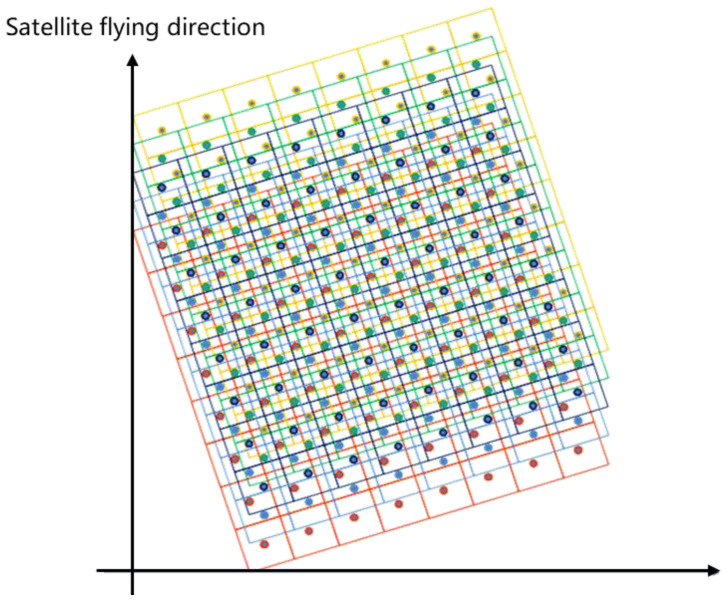
The diagram of oblique-mode sampling.

When $I_{1}$ = $I_{2}$ = … = $I_{N}$, MS-CMOS can realize planar array CMOS sensor function.

when α≠0, the sensor implements oblique-mode sampling, as shown in Figure 2.

## 4. MS-CMOS Imaging Simulation

The microsatellite with MS-CMOS sensor is scheduled to be launched this year. In the course of this research, the real MS-CMOS remote-sensing images cannot be obtained at the moment, the simulation function is given for the rotation angle sampling model of the sensor, which makes the simulation imaging process be realized by computer programming, simplifies the simulation process, and obtains the experimental data.

Since each row that forms the MS-CMOS is separately controlled, images simultaneously, and outputs independently, we design an imaging simulation mode of a single-line-array CMOS sensor.

**Figure 3:**
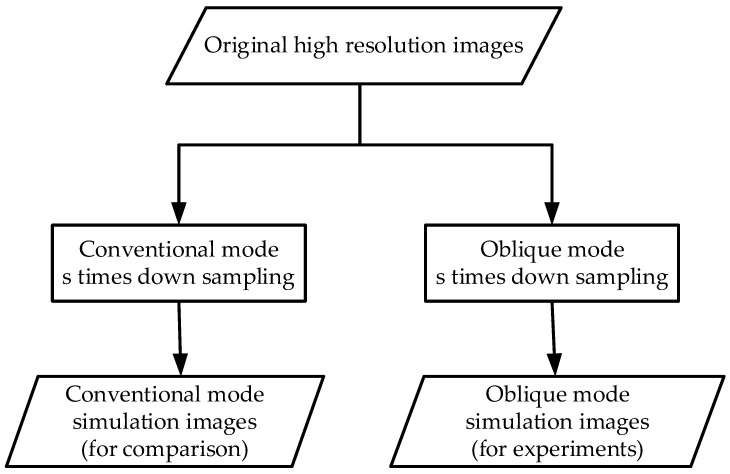
The flow chart of the imaging simulation mode.

The design of imaging simulation mode is based on the theory of image down-sampling. Down-sampling is an operation that reduces image resolution by using image interpolation algorithm to reduce image size. For an image which size is M·N, the resolution is reduced by S. times, and the image size is reduced by S. times. The flow chart of the imaging simulation mode is shown in Figure 3.

During the simulation process, the input HR images are sampled as LR images, we define a d-times down-sampling of the image, i.e., all the pixels in the d-d window are merged into a single pixel for output.

The linear array MS-CMOS sampling simulation mode is shown in function (2):

(2) $$I=F(\alpha ,A_{x},A_{y},d,m,p)$$

In this function:

I: MS-CMOS imaging model,

α: CMOS sensor rotation Angle,

$A_{x}$: abscissa of simulation starting point A(x,y)

$A_{y}$: ordinate of simulation starting point A(x,y)

*d*: down-sampling times,

m: pixel number of a linear array sensor,

*p*: sampling frequency of a linear array sensor, 

When α = 0, it becomes the conventional simulation mode.

**Figure 4:**
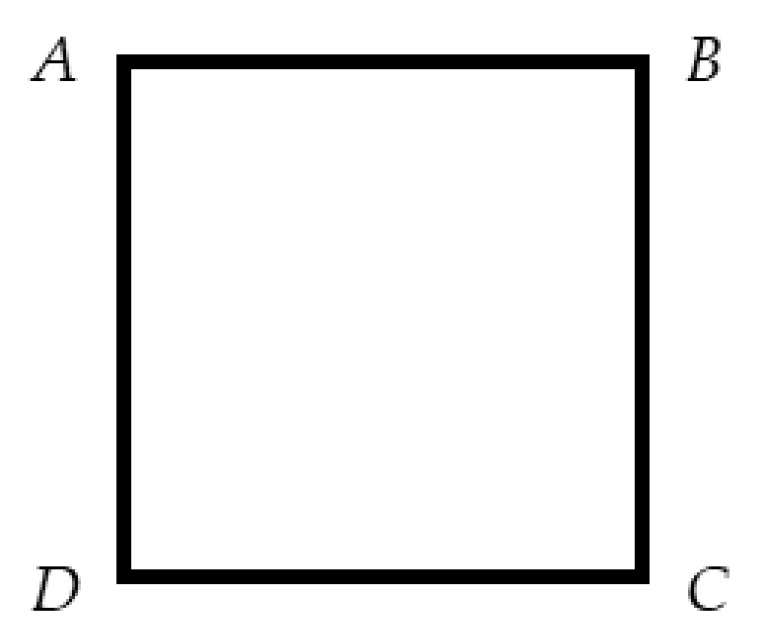
The diagram of four points.

We use $\tan\alpha =\frac{a}{b}$ stead of the angle α in the input, so the input values of the mode include m, p, d, a, b and the size of the input original HR image r×c. All the other variables in the following functions are process variables. Points A, B, C, D. form a down-sampling window in clockwise order, as shown in Figure 4.

Building the mode of the *j*th window in the *i*th imaging, which is shown in function (3):

(3) $$\{\begin{array}{c} m^{2}={\left(k\times a\right)}^{2}+{\left(k\times b\right)}^{2}\\ A_{xij}=\left(\left[k\times a\right]+1\right)+\left(i-1\right)\times \frac{d}{p}\\ A_{yij}=1\\ d^{2}={\left(n\times a\right)}^{2}+{\left(n\times b\right)}^{2}\\ B_{xij}=A_{xij}-n\times a\\ B_{yij}=A_{yij}+n\times b\\ C_{xij}=B_{xij}+n\times b\\ C_{yij}=B_{yij}+n\times a\\ D_{xij}=A_{xij}+n\times b\\ D_{yij}=A_{yij}+n\times a \end{array}$$

The loop function (4) is used in the simulation of the imaging down-sampling window:

(4) $$\{\begin{array}{c} A_{xi\left(j+1\right)}=B_{xij}\\ A_{yi\left(j+1\right)}=B_{yij}\\ B_{xi\left(j+1\right)}=A_{xi\left(j+1\right)}-n\times a\\ B_{yi\left(j+1\right)}=A_{yi\left(j+1\right)}+n\times b\\ C_{xi\left(j+1\right)}=B_{xi\left(j+1\right)}+n\times b\\ C_{yi\left(j+1\right)}=B_{yi\left(j+1\right)}+n\times a\\ D_{xi\left(j+1\right)}=C_{xij}\\ D_{yi\left(j+1\right)}=C_{yij} \end{array}$$

The simulation data can be obtained by traversing the whole original HR image.

## 5. Satellite-Using Super-Resolution Algorithm

Compared with the phenomenon that the effective resolution improvement ratio of the image processed by the existing SR methods is much smaller than the magnification of the image, the sequence images obtained by MS-CMOS in oblique-mode sampling contain high overlap and fixed dislocation information in the same area, which have more non-redundant information than that obtained by traditional optical satellites. First, the oblique mode can improve the resolution of the image in the satellite flight direction by increasing the sampling frequency. Secondly, sampling with any rotation angle realizes periodic cycle imaging, after the *n*th imaging, the image element of the (*n* + 1)th becomes superposition with that of the first imaging. The periodic overlap relations are used to reconstruct the SR image without registration, so that the resolution can be improved in the perpendicular direction. In this way, the SR process can be simplified, and more image information can be recovered.

The satellite-using SR algorithm can meet the requirements of the limited processing capacity and the limited available resources of the on-board computer, realize the SR reconstruction of multi-image and improve the satellite spatial resolution.

### 5.1. Parameters Correlation Analysis

According to the above mode, if we set: *n* is the minimum number of used MS-CMOS, α is the rotation angle, *f* is the sampling frequency, *d* is the spacing distance of sampling, S. is the times of resolution improvement (theoretical value), the relationship between sampling frequency and sampling spacing distance is shown in formula (5):

(5) $$f=\frac{1}{d}.$$

When the MS-CMOS is tilting an angle of α, the relationship between rotation angle and the minimum number of used MS-CMOS is shown in formula (6):

(6) $$\tan\alpha =\frac{1}{n}$$

At this point, the sensor sampling frequency and the sampling spacing distance meet the function (7) and (8):

(7) $$f=\sqrt{1+n^{2}}$$

(8) $$d=\frac{1}{f}=\frac{1}{\sqrt{1+n^{2}}}$$

During the imaging process, we use *n* rows of MS-CMOS to get *n* images. The SR processing of these images can increase the resolution of the images equally in both the satellite flight direction and the perpendicular direction, the increase time is:

(9) $$S=\sqrt{1+n^{2}}$$

For example, when tanα = 1, i.e., $\alpha =45^{\circ }$, at this time n=1, the satellite uses 1 row of MS-CMOS to get 1 image, the sampling frequency is $\sqrt{2}$ times of the original, the image resolution can be improved by $\sqrt{2}$ times (theoretical value).

When $\tan\alpha =\frac{1}{2}$, i.e., $\alpha =26.56^{\circ }$, at this time n=2, the satellite uses 2 rows of MS-CMOS to get 2 images, the sampling frequency is $\sqrt{5}$ times of the original, the image resolution can be improved by $\sqrt{5}$ times (theoretical value). 

### 5.2. Image Super-Resolution Reconstruction

Theoretically, MS-CMOS is capable of imaging at various angles. According to the requirements of the microsatellite design, this section discusses the algorithm of $26.56^{\circ }\left(\arctan\frac{1}{2}\right)$ rotation angle, and takes $26.56^{\circ }\left(\arctan\frac{1}{2}\right)$ as an example in the follow-up experiments.

**Figure 5:**
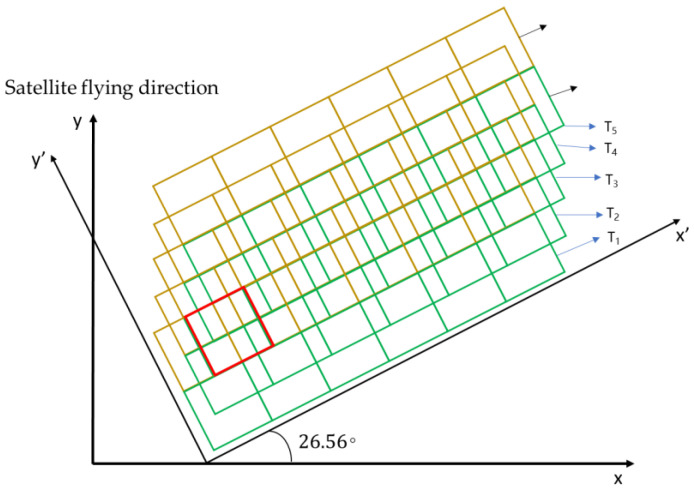
The diagram of 2 rows $26.56{}^{\circ}\left(\arctan\frac{1}{2}\right)$ oblique sampling mode.

According to the earlier discussion, when $\alpha =26.56^{\circ }$, the microsatellite uses 2 rows of MS-CMOS to get 2 images, the sampling frequency is $\sqrt{5}$ times of the original. The sampling mode is shown in Figure 5:

In Figure 5, 1 and 2 represent MS-CMOS row numbers, $T_{1}-T_{5}$ represent the imaging time. For a complete remote-sensing image obtained by splicing *N* strip images at *N* times, there exists pixel misplacement between rows in the image. The overlap rule is shown in formula (10):

(10) $$\{\begin{array}{c} \left(T_{M}{}_{x}-T_{\left(M+1\right)}{}_{x},T_{M}{}_{y}-T_{\left(M+1\right)}{}_{y})=(\frac{1}{4},\frac{1}{2}\right)\\ \left(T_{M}{}_{x}-T_{\left(M+2\right)}{}_{x},T_{M}{}_{y}-T_{\left(M+2\right)}{}_{y})=(\frac{1}{2},1\right) \end{array}$$

Secondly, the overlapping relationship between CMOS1 and CMOS2 is analyzed. Comparing the image obtained by CMOS2 at $T_{3}$ time with that obtained by CMOS1 at $T_{1}$ time, there is a 1/2 pixel overlap region of 0.5 pixel displacement in *x*’ direction and 0 pixel displacement in *y*’ direction, as shown in the red box of Figure 5. The pattern of dislocation and overlap between the images is shown in formula (11):

(11) $$\{\begin{array}{c} \mathrm{CMOS}1,T_{1}{}_{x},T_{1}{}_{y}=\mathrm{CMOS}2,\frac{1}{2}T_{3}{}_{x},T_{3}{}_{y}\\ \mathrm{CMOS}1,T_{2}{}_{x},T_{2}{}_{y}=\mathrm{CMOS}2,\frac{1}{2}T_{4}{}_{x},T_{4}{}_{y}\\ \mathrm{CMOS}1,T_{3}{}_{x},T_{3}{}_{y}=\mathrm{CMOS}2,\frac{1}{2}T_{5}{}_{x},T_{5}{}_{y}\\ \ldots \\ \mathrm{CMOS}1,T_{\left(N-2\right)}{}_{x},T_{\left(N-2\right)}{}_{y}=\mathrm{CMOS}2,\frac{1}{2}T_{N}{}_{x},T_{N}{}_{y} \end{array}$$

According to the two kinds of overlapping patterns, the SR reconstruction result is recorded as *H*, the CMOS1 imaging is $L_{1}$, and the CMOS2 imaging is $L_{2}$. The reconstruction model is shown in formula (12):

(12) $$H(i,j)=\left(L_{1}\left(i+1,j+1\right)+L_{2}\left(i,j\right)\right)/2$$

$H\left(i,j\right),\;L_{1}\left(i,j\right)$ and $L_{2}\left(i,j\right)$: The gray value of the pixel in (i,j) position.

### 5.3. Image Deformation Correction

Images sampled in oblique mode are distorted due to the image storage and display mechanism of the computer. Oblique sampling makes the real data change from the traditional rectangle to the parallelogram with jagged edges, and the images in the computer are automatically arranged into a rectangle display, which results in the deformation of the images.

Deformation correction is the inverse transformation of rotation angle, which means the coordinate transformation of each pixel in the image. The super-resolution image is recorded as *SR*, *H* has the same meaning as above, the inverse transformation mode is shown in formula (13) and (14):

(13) SR =F(α,H)

Specifically:

(14) $$\{\begin{array}{c} SR_{x}=\alpha \times H_{x}\\ SR_{y}=H_{y} \end{array}$$

In this function:

$SR_{x},\;SR_{y}$: abscissa and ordinate of pixel (i,j) in SR image,

α: MS-CMOS rotate angle,

$H_{x},H_{y}$: abscissa and ordinate of pixel (i,j) in SR reconstruction result.

## 6. Experiments and Discussion

### 6.1. Simulation Experiments

There are two types of simulation data. The first kind of data is the experimental data of satellite-using SR algorithm. We use the simulation algorithm to conduct the down-sampling operation with the resolution of the original HR image decreased by 10 times. Specifically, we simulate a 2-rows MS-CMOS with an angle of $26.56^{\circ }\left(\arctan\frac{1}{2}\right)$, $\sqrt{5}$ times of the frequency sampling. The second kind of data is used as the comparison data of the experimental results of the algorithm, which simulates the conventional sampling of microsatellite, we sample the original HR image with a resolution reduction of 10 times.

**Figure 6:**
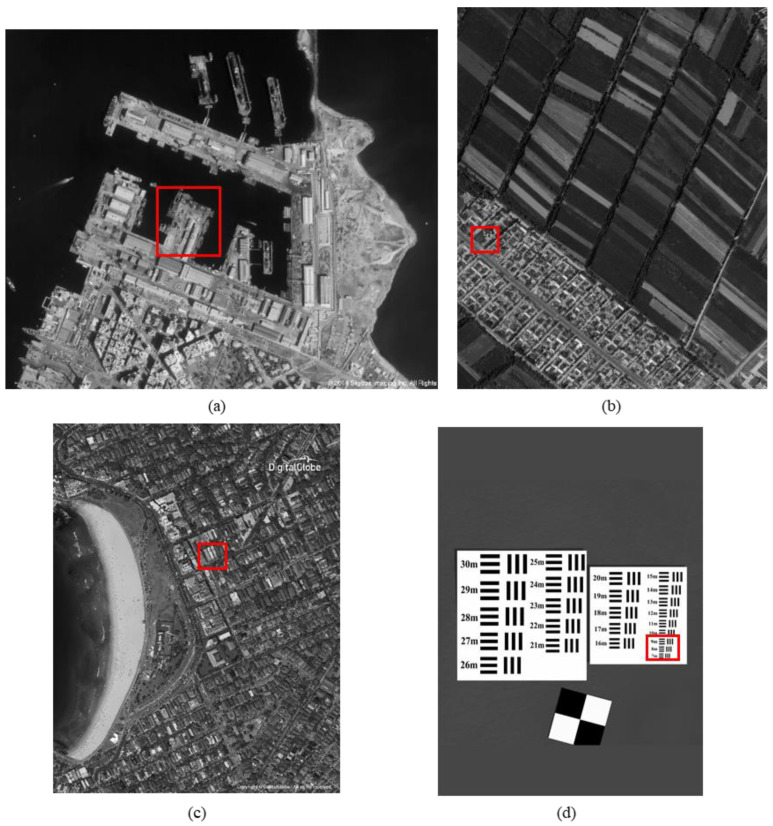
The original high-resolution images: (**a**) seashore remote-sensing image, the chosen area is an offshore structure in an Egyptian port; (**b**) farmland remote-sensing image, the selected area is the crops and houses in Xinjiang, China; (**c**) city remote-sensing image, the selected area is buildings in Sydney; (**d**) target image, the selected area is minimum resolvable line pairs.

**Figure 7:**
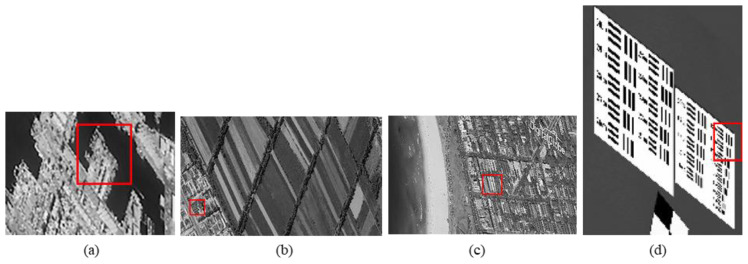
CMOS1 oblique-mode sampling simulation images: (**a**) seashore remote-sensing image, the chosen area is an offshore structure in an Egyptian port; (**b**) farmland remote-sensing image, the selected area is the crops and houses in Xinjiang, China; (**c**) city remote-sensing image, the selected area is buildings in Sydney; (**d**) target image, the selected area is minimum resolvable line pairs.

**Figure 8:**
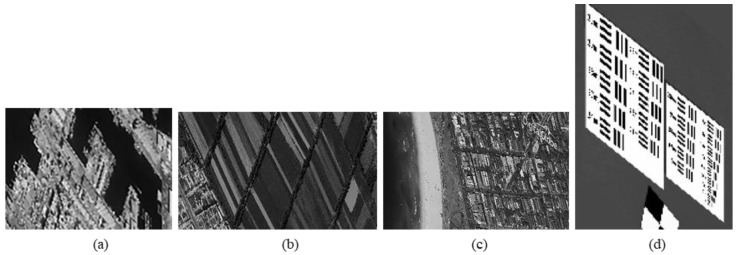
CMOS2 oblique-mode sampling simulation images: (**a**) seashore remote-sensing image; (**b**) farmland remote-sensing image; (**c**) city remote-sensing image; (**d**) target image.

**Figure 9:**
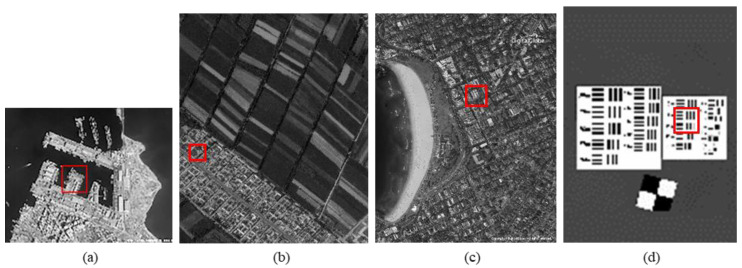
MS-CMOS conventional mode sampling simulation images: (**a**) seashore remote-sensing image, the chosen area is an offshore structure in an Egyptian port; (**b**) farmland remote-sensing image, the selected area is the crops and houses in Xinjiang, China; (**c**) city remote-sensing image, the selected area is buildings in Sydney; (**d**) target image, the selected area is minimum resolvable line pairs.

The original HR images selected for the simulation include one SkySat-2 image (seashore remote-sensing image with a resolution of 0.8 m), two Worldview-4 images (farmland and city remote-sensing images with a resolution of 0.31 m) and one target image (with a resolution of 2 m). These images are rich in texture details, which can provide a good reference for the following experiments. Figure 6 shows the original data, Figure 7 and Figure 8 show the experimental data, Figure 9 shows the comparison data. The areas selected by the red box in each type of the images are the same, those local images will be enlarged in Section 6.3 and Section 6.4 to analyze the details.

### 6.2. Algorithm Experiments

**Figure 10:**
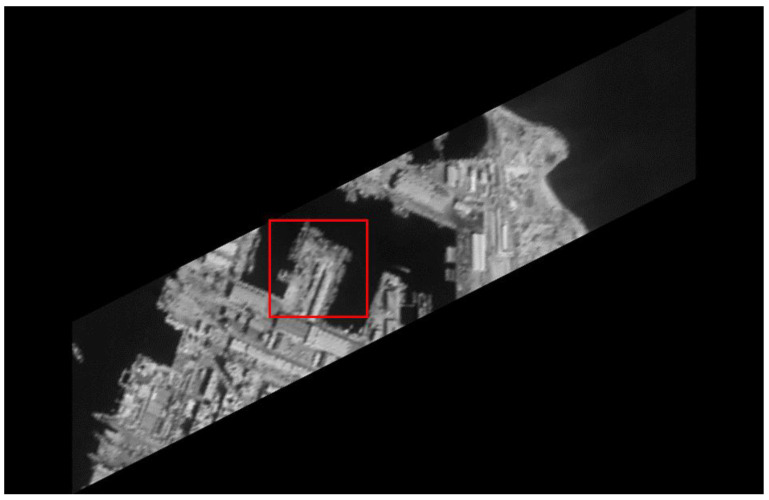
The seashore remote-sensing image experimental results: super-resolution images, the chosen area is an offshore structure in an Egyptian port.

**Figure 11:**
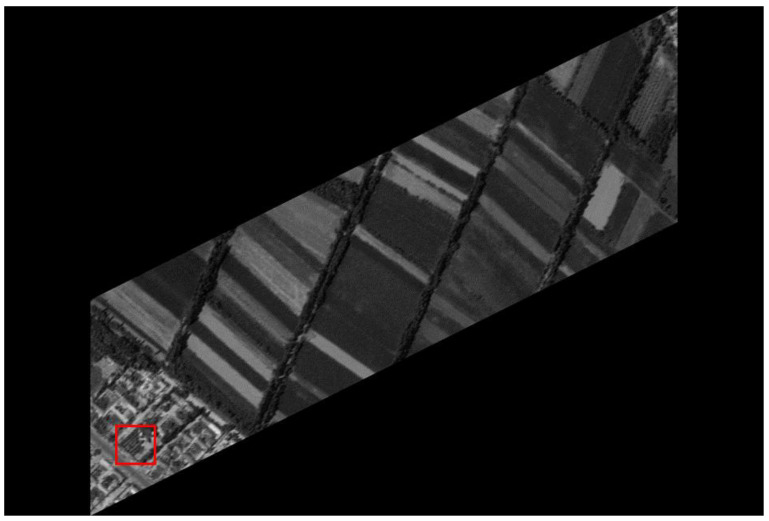
The farmland remote-sensing image experimental results: super-resolution images, the selected area is the crops and houses in Xinjiang, China.

**Figure 12:**
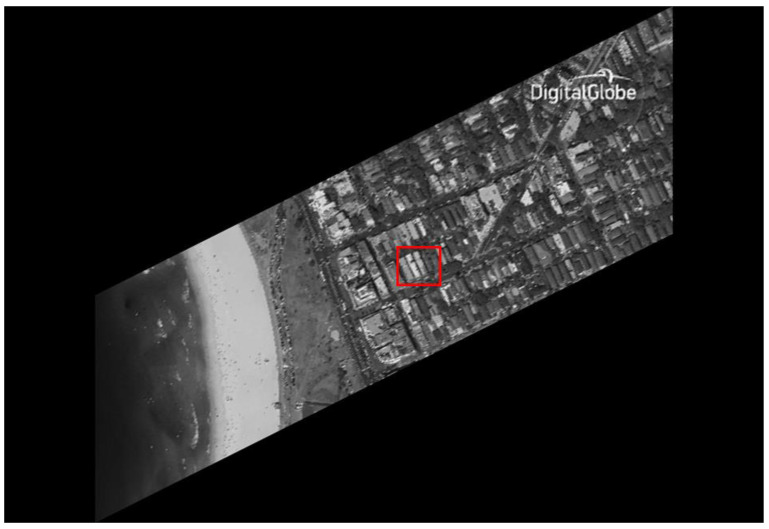
The city remote-sensing image experimental results: super-resolution images, the selected area is buildings in Sydney.

**Figure 13:**
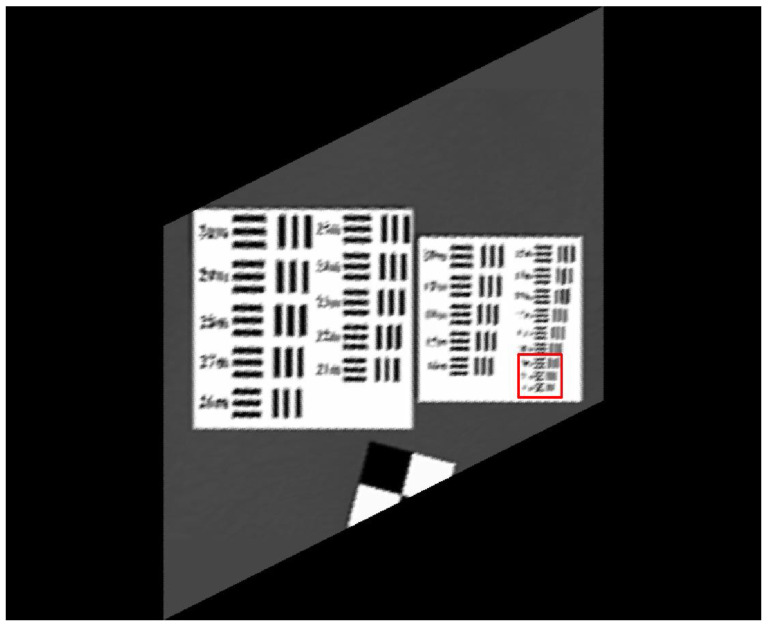
The target remote-sensing image experimental results: super-resolution images, the selected area is minimum resolvable line pairs.

We experiment the $26.56^{\circ }\left(\arctan\frac{1}{2}\right)$ algorithm by using MATLAB (Matrix & Laboratory software) R2016a. The experimental content includes two parts: image SR reconstruction and image deformation correction. Figure 10 shows the seashore SR image, Figure 11 shows the farmland SR image, Figure 12 shows the city SR image, Figure 13 shows the target SR image. The areas selected by the red box in each type of the images are the same, those local images will be enlarged in Section 6.3 and Section 6.4 to analyze the details.

### 6.3. Evaluation of Super-Resolution Image Quality

**Figure 14:**
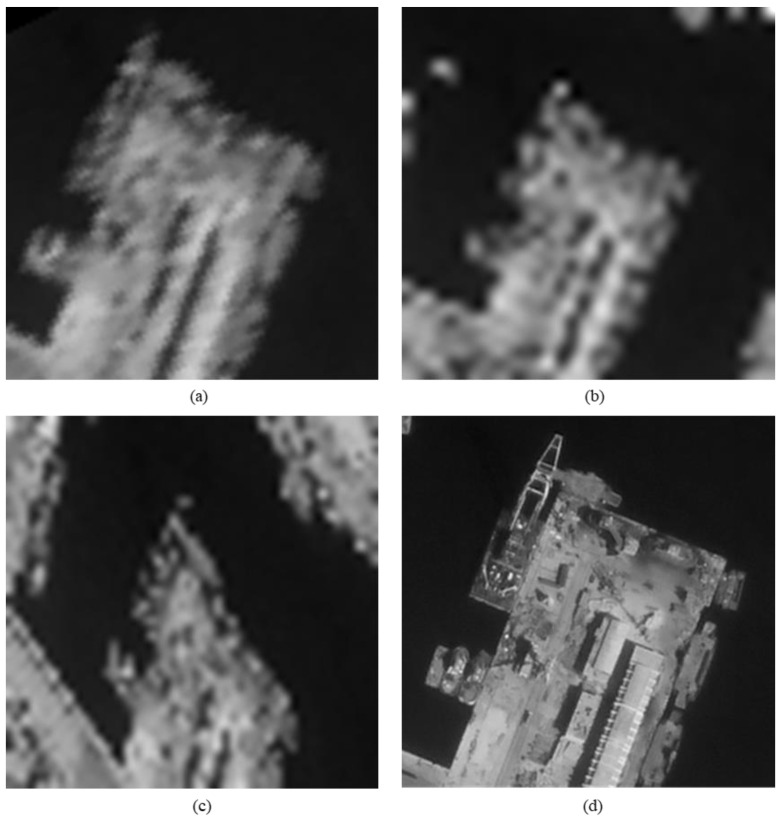
The local magnification of the seashore remote-sensing image: (**a**) super-resolution image, (**b**) conventional mode simulation image, (**c**) CMOS1 oblique-mode simulation images, (**d**) original high-resolution image.

**Figure 15:**
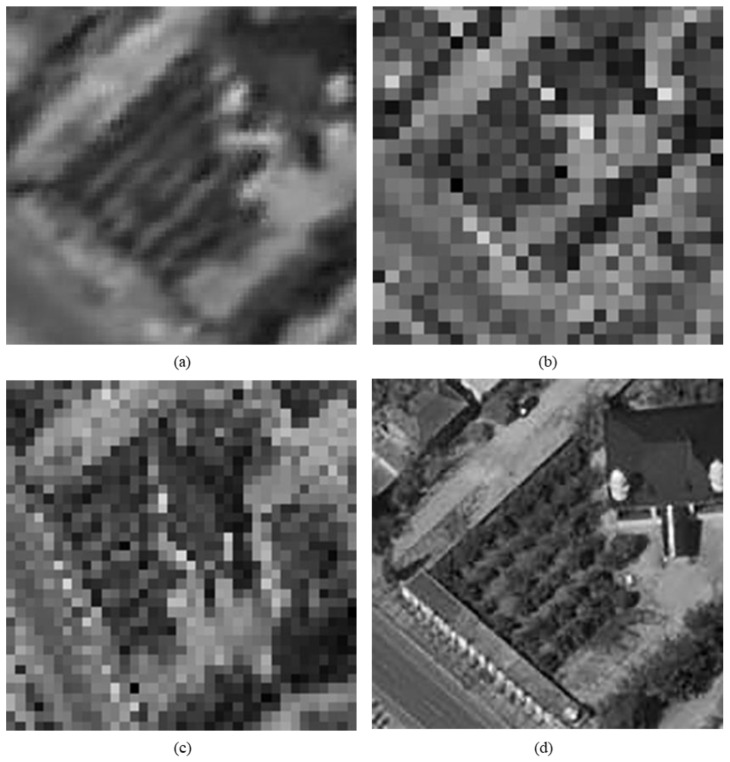
The local magnification of the farmland remote-sensing image: (**a**) super-resolution image, (**b**) conventional mode simulation image, (**c**) CMOS1 oblique-mode simulation images, (**d**) original high-resolution image.

**Figure 16:**
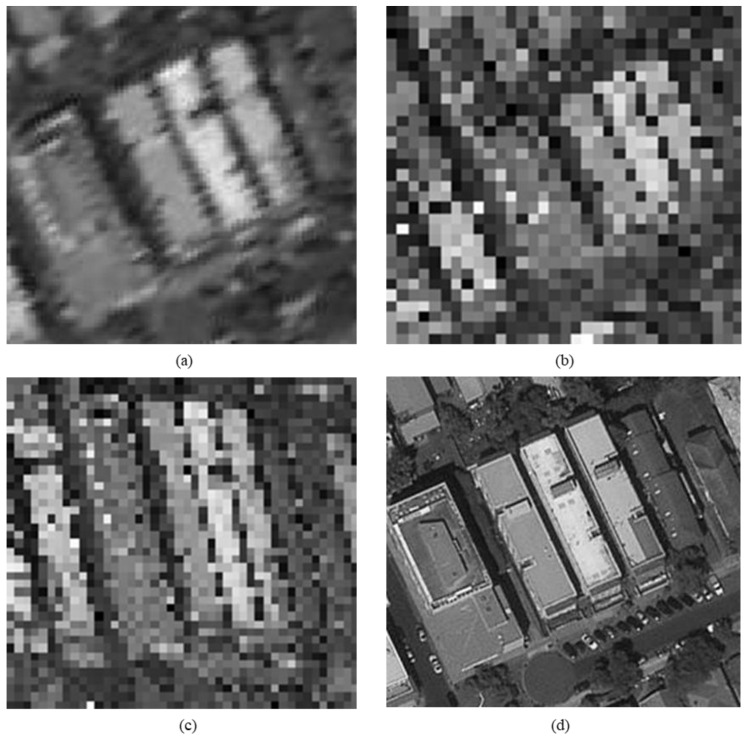
The local magnification of the city remote-sensing image: (**a**) super-resolution image, (**b**) conventional mode simulation image, (**c**) CMOS1 oblique-mode simulation images, (**d**) original high-resolution image.

In this section, we discuss the enlarged red box selected areas of the simulation data and the experimental results. Figure 14 shows the local magnification images of the seashore image in Figure 10, Figure 9, Figure 6, and Figure 7 in turn. Figure 15 shows the local magnification images of the farmland image in Figure 11, Figure 9, Figure 6, and Figure 7 in turn. Figure 16 shows the local magnification images of the city image in Figure 12, Figure 9, Figure 6, and Figure 7 in turn. 

In the experiment, we select areas with rich texture and distinct features to enlarge, and analyze the ability of this algorithm in restoring the edge and other details. Figure 14 shows the chosen area of the seashore image which is an offshore structure in an Egyptian port, there is a distinct contour feature in the upper left corner of Figure 14d. Figure 14b lost the contour information in the upper left corner. However, Figure 14a, processed by the algorithm, successfully recovers part of the contour information, which is more similar to Figure 14d the original image. Figure 15 shows the chosen area of the farmland image which is the crops and houses in Xinjiang, China. The area contains an orderly array of crops and buildings, the crops in this area have distinct texture features, as shown in Figure 15d. In Figure 15b, the image information is indistinguishable, only fuzzy boundaries can be seen. However, Figure 15a, processed by the algorithm, not only contains the boundary, but also has parts of the crops texture information and roof features, which is more similar to Figure 15d the original image. Figure 16 shows the chosen area of the city image which is buildings in Sydney. The area contains distinct contour and edge features, as shown in Figure 16d. In Figure 16b, the edges are indistinguishable, and the feature information is lost. However, in Figure 16a, processed by the algorithm, buildings can be well distinguished, and the image retains some contour and edge information, which is more similar to Figure 16d the original image.

In addition, comparing the image (b) and the image (c) in Figure 14, Figure 15 and Figure 16, the images obtained by oblique-mode sampling are richer in edge details than those obtained by conventional mode sampling, which indicates that the special imaging design of MS-CMOS has improved the image resolution to a certain extent.

### 6.4. Evaluation of Algorithm Effectiveness

The effectiveness evaluation of the algorithm is to evaluate the ability of improving the image resolution. In this paper, the improvement of target image resolution is calculated to realize evaluation.

The target image usually consists of pairs of lines of different thickness, with different line pairs representing different resolution sizes. We evaluate the improvement times of image resolution by determining the corresponding value of the limit resolvable line pairs of the target image (minimum resolvable contras).

Let *x* represent the minimum value of the three lateral lines in the target image which can be resolved to the limit, let y represent the minimum value of the three vertical lines in the target image which can be resolved to the limit, the calculation formula of target image resolution is:

(15) $$Target\;Resolution=\sqrt{\frac{x^{2}+y^{2}}{2}}$$

We enlarge the red box of the target image in Section 6.1 and Section 6.2, calculate the resolution of each target image.

**Figure 17:**
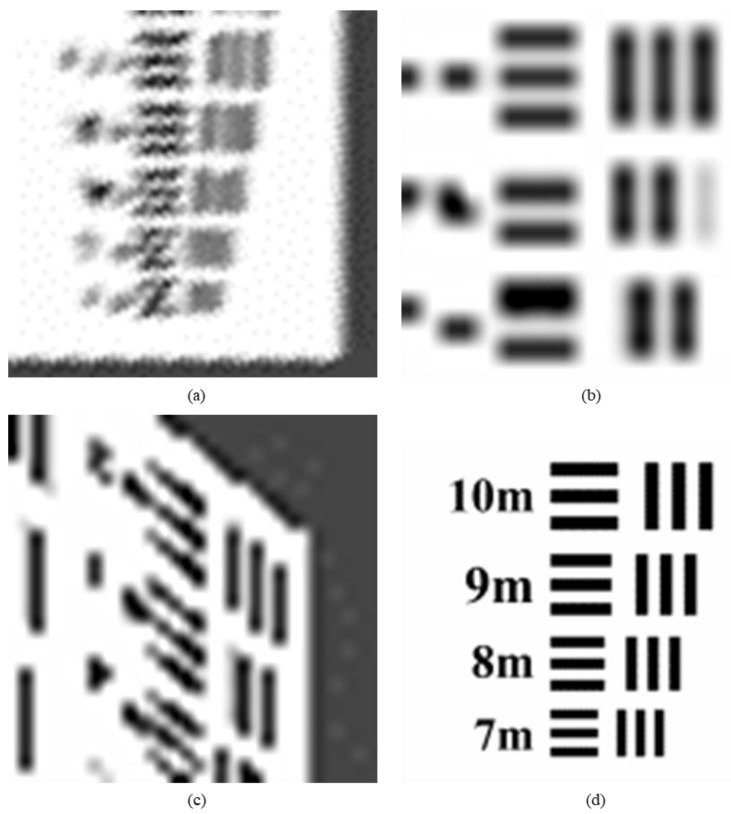
The local magnification of the target remote-sensing image: (**a**) super-resolution image, (**b**) conventional mode simulation image, (**c**) CMOS1 oblique-mode simulation images, (**d**) original high-resolution image.

Figure 17 shows the selected area of target image which is the minimum resolvable line pairs. The image resolution of the original target image (Figure 17d) is 2 m. From the image (b) in Figure 17, the minimum resolvable value of line pairs is 19 m, according to the formula (15), the target resolution represented in Figure 17b is 19.00 m. From the image (c) in Figure 17, the minimum resolvable value of lateral line pairs is 13 m, the minimum resolvable value of vertical line pairs is 15 m, according to the formula (15), the target resolution represented in Figure 17c is 14.03 m. It confirms the conclusion of the previous section on the subjective evaluation of other experimental results, the resolution of MS-CMOS oblique-mode sampling data has been improved compared with the conventional sampling of sensors.

From the image (a) in Figure 17, the minimum resolvable value of line pairs is 9 m, according to the formula (15), the target resolution represented in Figure 17 image (a) is 9.00 m. Compared with the conventional sampling image, the resolution of SR image is increased by $\frac{19.00}{9.00}\approx 2.11$ times through the processing method of MS-COMS combined with the algorithm. The result is close to the theoretical value of $\sqrt{5}$ times, which proves that the proposed algorithm is effective.

### 6.5. Evaluation of Algorithm Practicality

Since the rotation of the satellite sensor cannot be accurately transferred to the theoretical design value of $26.56^{\circ }$, this section discusses the influence of rotation error on image resolution improvement. We rotate the original image of the target every $10^{\circ }$ (rotate $180^{\circ }$ clockwise and $180^{\circ }$ counterclockwise respectively) to obtain 36 new target images, in which the anticlockwise rotation is represented by a negative value, repeat the experiment process and calculate the resolution. We calculate the lateral line pairs, the vertical line pairs and the image resolution improvement of the target image after clockwise rotation and counterclockwise rotation respectively, and the results are shown in the tables.

In Table 1, when the data is rotated clockwise, the improvement of image resolution is more influenced by the limit resolvable scale of the vertical line pairs. The minimum value of the image resolution improvement appears at $140^{\circ }$, which is 1.51 times. The complementary angle of $26.56^{\circ }$ is the fourth quadrant angle, which is the clockwise $153.44^{\circ }$. The data around this angle, which are $140^{\circ }\sim 160^{\circ }$ images, the times of vertical resolution improvement of these data all show local minimum values. This means that when the angle between the actual orbit and the predetermined orbit appears clockwise due to the attitude adjustment or yaw of the satellite, when the algorithm is actually operated on the microsatellite, the spatial resolution can be improved at least 1.51 times.

In Table 2, when the data is rotated counterclockwise, the improvement of image resolution is more influenced by the limit resolvable scale of lateral line pairs. The minimum value of the image resolution improvement appears at $-140^{\circ }$, which is 1.41 times. The redundancy angle of $26.56^{\circ }$ is the third quadrant angle, which is the counterclockwise $116.56^{\circ }$ ($-116.56^{\circ }$). The data around this angle, which are $-120^{\circ }\sim -150^{\circ }$ images, the times of lateral resolution improvement of these data all show local minimum values. This means that when the angle between the actual orbit and the predetermined orbit appears counterclockwise due to the attitude adjustment or yaw of the satellite, when the algorithm is actually operated on the microsatellite, the spatial resolution can be improved at least 1.41 times.

Based on the above analysis, it can be seen that there are differences in the resolution improvement times of the images which are used for the algorithm experiment after being rotated. When the satellite rotates clockwise, the image resolution of the target image increases by 1.78 times on average, with a minimum increase of 1.51 times; when the satellite rotates counterclockwise, the image resolution of the target image increases by 1.80 times on average, with a minimum increase of 1.41 times.

Therefore, the algorithm satisfies the preset technical index (the spatial resolution is increased by $\sqrt{2}\approx 1.41$ times) when the project was designed, the algorithm has on-board practicality.

### 6.6. Evaluation of Algorithm Efficiency

The spaceborne data acquisition and processing system is an embedded system which is suitable for real-time and multi-task processing. Its main functions include collecting, framing, and transmitting the satellite telemetry information, executing the indirect remote control command, measuring, and controlling the satellite attitude, correcting the orbit autonomous fault, diagnosis and processing of satellite, and satellite missions and resources management. The hardware part mainly includes a FPGA (Field Programmable Gate Array) chip, a spaceborne computer, storage equipment, and so on. The software part includes pre-embedded development image processing programs, image compression programs, storage file systems, and so on. Some indicators of the satellite-borne data acquisition and processing system are as follows:

- Microsatellite storage space: 1 TB
- Data transmission capacity: 450 Mbps
- Pixel size of MS-CMOS sensor: 7.4 µm × 7.4 µm
- Pixel number of MS-CMOS sensor: 5144 × 3800
- FPGA type: XILINX UltraScale
- FPGA computing power: 3.15 Tflops (0.7 × 4.5 Tflops (theoretical maximum value))
- HP (Hewlett-Packard) desktop computing power: 211.2 Gflops

The ground running environment of the algorithm is a HP desktop and MATLAB R2016a software. The algorithm comes from the theory of image interpolation. Besides the function of image reading, display, and storage, the core code of the program uses the basic image pixel operation and geometric transformation operation in MATLAB, and the method is simple. The experimental data obtained by simulation is smaller than the real remote-sensing images. We enlarge the experimental data to approximate the size of the microsatellite images for evaluation, the results are shown in Table 3. The program runs with less cache data, less memory, high efficiency, and short time. The time used includes the length of the image display function. When the algorithm is transplanted to the microsatellite computer, the image display function will not be needed. The actual running time will be further shortened. Because the FPGA chip has more computing power than the HP desktop, the proposed algorithm can adapt to the limited computer resource environment of the satellite, the algorithm has a certain satellite-using efficiency.

## 7. Conclusions

In this paper, a simple and efficient microsatellite-using SR algorithm is proposed for multi-modality super-CMOS sensor. First, the spatial resolution is improved from the hardware design by using the oblique sampling mode of the MS-CMOS sampling at several times of the conventional sampling frequency. Secondly, based on the displacement relationship between the oblique pattern sequences, the image SR reconstruction model and distortion correction is established, and several LR images are synthesized into one HR image to improve the spatial resolution. It is proved that the algorithm can effectively improve the satellite resolution, meet the preset engineering indexes, and have the feasibility of operating on the satellite.

Due to the limited resources on the microsatellite, complex algorithms cannot be realized. With the improvement of microsatellite technology, image preprocessing and other operations will be completed on the satellite, so that the image will get better results. When the data is transmitted to the ground, the resolution can be further improved, and the image information can be enriched by combining with other SR algorithms.

## Figures and Tables

**Table 1:** Comparison of clockwise rotation resolution improvement times.

Angle	Times of Lateral Resolution Improvement	Times of Vertical Resolution Improvement	Times of Image Resolution Improvement
0°	2.11	2.11	2.11
10°	1.90	1.82	1.86
20°	1.67	1.82	1.74
30°	1.74	2.11	1.89
40°	1.50	1.70	1.59
50°	1.50	2.25	1.77
60°	1.46	2.11	1.70
70°	1.29	2.38	1.62
80°	1.73	2.38	1.98
90°	1.90	2.38	2.10
100°	1.90	2.00	1.95
110°	2.00	1.89	1.95
120°	2.13	1.45	1.72
130°	2.25	1.50	1.77
140° 150° 160°	1.78 2.25 2.00	1.331.381.38	1.51 1.67 1.61
170°	1.58	1.50	1.54
180°	2.11	1.63	1.84
**Average**	**1.83**	**1.85**	**1.78**

**Table 2:** Comparison of counterclockwise rotation resolution improvement times.

Angle	Times of Lateral Resolution Improvement	Times of Vertical Resolution Improvement	Times of Image Resolution Improvement
0°	2.11	2.11	2.11
−10°	2.11	1.82	1.94
−20°	2.50	1.75	2.01
−30°	2.38	1.38	1.71
−40°	2.00	1.31	1.57
−50°	2.11	1.36	1.61
−60°	2.11	1.55	1.79
−70°	2.25	2.38	2.31
−80°	2.00	1.90	1.95
−90°	1.80	1.90	1.85
−100°	1.73	2.00	1.84
−110°	1.58	2.25	1.81
−120° −130° −140° −150°	1.381.211.211.29	2.25 1.80 1.78 1.80	1.67 1.44 1.41 1.48
−160°	1.90	2.00	1.95
−170°	1.90	1.80	1.85
−180°	2.11	1.64	1.84
**Average**	**1.88**	**1.83**	**1.80**

**Table 3:** Algorithm running time.

Name	Image Size	Time (s)
Seashore image	4142 × 5661 (8.03 M)	0.852
Farmland image	2628 × 3240 (3.28 M)	0.679
City image	2928 × 3762 (6.20 M)	0.748
Target image	1024 × 1024 (1.00 M)	0.584
**Average**	**4.63 M**	**0.713**
